# Novel Hydrophobic Associating Polymer with Good Salt Tolerance

**DOI:** 10.3390/polym10080849

**Published:** 2018-08-01

**Authors:** Jincheng Mao, Hongzhong Tan, Bo Yang, Wenlong Zhang, Xiaojiang Yang, Yang Zhang, Heng Zhang

**Affiliations:** State Key Laboratory of Oil and Gas Reservoir Geology and Exploitation, Southwest Petroleum University, Chengdu 610500, China; 201621000738@stu.swpu.edu.cn (H.T.); 201511000098@stu.swpu.edu.cn (X.Y.); 201521000098@stu.swpu.edu.cn (Y.Z.); 201621000198@stu.swpu.edu.cn (H.Z.)

**Keywords:** hydrophobic associating polymer, salt tolerance, mechanism

## Abstract

A hydrophobic associating polymer named DiPHAM (acrylamide/sodium acrylamide-2-methylpropanesulfonic/sodium acrylate/*N*,*N*-di-*n*-dodecylacrylamide) with good salt tolerance was synthesized via photo-initiation polymerization. The critical association concentration (CAC) of DiPHAM was determined by viscosity changes to be 490 mg/L with different DiPHAM concentrations and particle sizes varied under such dynamic conditions. The influences of aqueous metal ions with different charges on its aqueous solution were investigated by measuring apparent viscosity, viscoelasticity, thixotropy, rheology, and particle size, and by SEM observation. The apparent viscosity of the DiPHAM solution was affected by metal ions to some extent, but the viscosity of the polymer can be still maintained at 55 mPa·s under 20 × 10^4^ mg/L NaCl. Divalent metal ions show greater impact on DiPHAM aqueous solutions, but the polymer solutions showed resistance to the changes caused in viscosity, structure, and viscoelasticity by Ca^2+^ and Mg^2+^ ions. The salt tolerance of DiPHAM is due to the combination of hydrophobic association, the electrostatic shield, and double layer compression of the hydration shell. Increasing the ion concentration enhances the dehydration and further compresses the hydration shell, making the non-structural viscosity decrease, even “salting out”. Measurements of rheological properties showed that DiPHAM solutions could maintain a relatively high viscosity (0.6%-71 mPa·s/0.3%-50 mPa·s) after 120 min of continuous shearing (170 s^−1^) at 140 °C. Under high-salinity (5000 mg/L Ca^2+^/3000 mg/L Mg^2+^) conditions, the solution with 0.6 wt% DiPHAM still maintained a high viscosity (50 mPa·s/70 mPa·s) after continuously shearing for 120 min at 120 °C and 170 s^−1^. The good salt tolerance of DiPHAM can lead to a variety of applications, including in fracturing fluids for enhanced oil recovery (EOR) and in sewage treatment.

## 1. Introduction

Water-soluble polymers with a small number of hydrophobic groups in their main chains are called hydrophobic associating polymers. Due to their properties in aqueous solution, which are caused by the interaction of intramolecular and intermolecular chains between the hydrophobic groups, they are widely used in oil and gas development, engineering materials, industrial coatings, sewage sludge, and drug controlled release [1,2], but their properties and structures can be greatly affected by salinity and temperature. Most studies have only revealed the effect of salinity on the viscosity of polymer solutions, but the effects on the microstructure and other rheological properties have rarely been reported [3,4].

McCormick found that the introduction of hydrophobic monomers in polymers could increase the hydrodynamic volume of the polymers in aqueous solution and give the polymers a better salt resistance [5]. Klucker used static and dynamic light scattering (DLS) to show that hydrophobic association polymers will form a network structure in aqueous solution, leading to salt resistance [6]. Yamamoto and co-workers studied sodium acrylamide-2-methylpropanesulfonic (AMPS-Na)/methyl acrylamide alkyl substituted random copolymer and showed that an increase in the number of carbon atoms in the long hydrophobic chains leads to enhanced polymer association [7]. By studying the transfection efficiency of amphiphilic polymer to genes, Ying found that a longer hydrophobic chain length leads to better self-assembly efficiency [8,9]. Shalaby et al. found that polymers containing –COO^−^, –SO_3_^−^, –RCOO^−^ and other anionic groups have very obvious polyelectrolyte adhesion [10]. Dusseault synthesized a salt-resistant polymer (KY700) for oil and gas field development, whose viscosity stays at 97% with 4500 mg/L mineralization [11]. Jiang has prepared a nonionic modified polyacrylamide of P(AM/OP-10-AC), whose aqueous solution can maintain good rheological properties in 6 wt% of KCl [12]. Zhu developed a hydrophobic associating polymer for which a 0.15 wt% solution can maintain 50% of its viscosity with 10,000 mg/L NaCl [13]. Sarsenbekuly prepared a hydrophobic associating polymer (RH-4) that can maintain high shear viscosity in 80,000 mg/L NaCl aqueous solutions and has an excellent tolerance to Ca^2+^ and Mg^2+^ ions due to its great hydrophobic association performance [14].

In this work described in this paper, an anionic hydrophobic association polymer named DiPHAM was designed and synthesized for use in high-salinity water. The design called for using acrylamide as the main polymerization monomer, and the commonly used and widely available salt-resistant anionic monomer AMPS-Na was introduced into the polymer chain. At the same time, in order to further enhance the salt resistance, a twin-tailed hydrophobic monomer *N,N*-di-*n*-dodecylacrylamide (DiC_12_AM), which has stronger thickening properties due to a stronger hydrophobic effect, was also introduced into the polymer. In addition, a hydrophilic anionic monomer sodium acrylate (AA-Na) was introduced with a purpose to improve solubility. 

The particle sizes of the polymer were measured by dynamic light scattering (DLS) to ascertain the critical association concentration (CAC) of DiPHAM. The changes in the polymer before and after association and the changes in other characteristics in salt solutions with different concentrations were observed to analyze the salt-tolerance mechanism of the polymer [15]. This paper not only describes the changes in the viscosity of the polymer in various salt solutions, but also describes other properties of polymer solutions with high salinity, including shearing resistance, thixotropy, viscoelasticity, and other rheological properties. The results show that DiPHAM could be used extensively, maintaining stable structural properties in the high-salinity solutions.

## 2. Materials and Experiment

### 2.1. Raw Materials

Raw materials of acrylamide (AM, Kelong, Chengdu, China), 2-acrylamido-2-methylpropane sulfonate (AMPS, Best-reagent, Chengdu, China), acrylic acid (AA, Kelong, Chengdu, China), methacryloylchloride (Kelong, Chengdu, China), 2,2′-azobis (2-methylpropionamidine) (Best-reagent, Chengdu, China), dihydrochloride (V50, Kelong, Chengdu, China), dodecyl amine, bromo dodecane (Kelong, Chengdu, China), sodium dodecyl sulfate (SDS, Kelong, Chengdu, China), sodium chloride (Kelong, Chengdu, China) and sodium hydroxide (Kelong, Chengdu, China) were analytical reagent grade and were purchased from chemical markets without further purification. Twin-tailed hydrophobic monomer DiC_12_AM was prepared as described in the literature [16].

### 2.2. Synthesis of DiPHAM

**Scheme 1:**
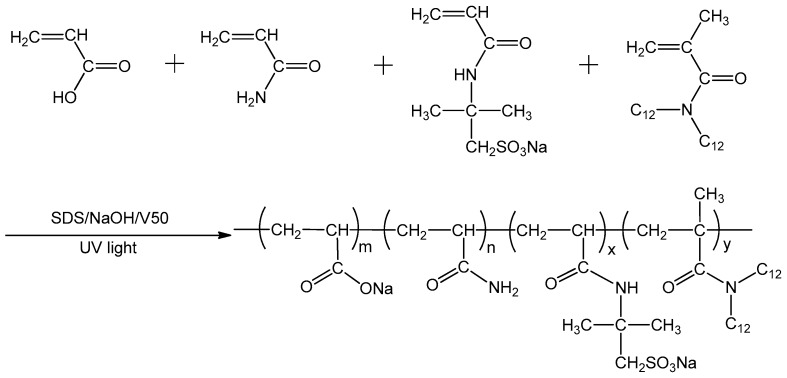
The synthesis of DiPHAM.

The reaction was conducted in a three-necked flask and equipped with a magnetic stirrer. A certain amount of AM (28.26 g), AA (10.31 g), AMPS (5.84 g), DiC_12_AM (0.47 g) and SDS (6.58 g) were weighed precisely (Scheme 1), the molar ratio of SDS to hydrophobic monomer was 20, and dissolved in deionized water to form a clarified mixed solution, and then NaOH solution (30 wt%) was added until the solution had reached a pH of 6 to 7 [17].

Nitrogen was bubbled through a three-necked flask for 30 min to ensure the reaction was conducted in an inert atmosphere. A certain amount of V50 (0.1 wt%) was added into solution as the initiator after the hydrophobic monomer had dissolved completely, and then the flask was placed in a UV light fixture. Polymer colloid was obtained via illumination reaction after 6 h at 25 °C. The colloid was then cut into small pieces and precipitated with anhydrous ethanol for several times. Finally, hydrophobic polymer product was acquired after vacuum drying and granulation.

## 3. Methods

### 3.1. Characterization

The Fourier transform infrared (FT-IR) spectrum of granulated DiPHAM was acquired by an Infrared spectrometer (Beijing Rayleigh Analytical Instrument Corporation, Beijing, China, WQF-520), using the potassium bromide disk method. The spectrum was obtained at a resolution of 0.5 cm^−1^. The nuclear magnetic resonance hydrogen spectrum (^1^H NMR) of DiPHAM was obtained with a Bruker 400 MHz NMR spectrometer (Bruker AVANCE III HD 400, Karlsruhe, Germany), operating at 400 MHz. The DiPHAM was prepared in D_2_O with a concentration of 10 g/L after being soaked with acetone for 24 h to remove impurities.

### 3.2. DLS Measurements

The hydrodynamic radius (*R*_g_) of a polymer can indicate its particle size and the size of polymer aggregates in aqueous solutions. The particle size of DiPHAM was determined by dynamic light scattering with a wide angle laser light scatterometer (Brookhaven, BI-200SM, Suffolk, NY, USA). DiPHAM was dissolved in deionized water to prepare the polymer solutions. The polymer salt solutions were prepared by adding NaCl to 500 mg/L DiPHAM solutions. The temperature for the tests was 25 °C, the laser module was a 532-Na light source, the detection angle was 90°, and CONTIN software was used for the final analysis of data.

### 3.3. Rheological Properties Evaluation

Rheological properties, including shear-induced behavior, shear resistance, viscoelasticity and thixotropy, are the most critical indicators for evaluating fluids. The temperature resistance and shear resistance are important reference indicators for polymer applications. The rheological properties of DiPHAM solutions were investigated using a HAAKE MAR III RS 600 rheometer (Haake, Karlsruhe, Germany) equipped with a high-pressure sealed cell.

### 3.4. Microstructure Analysis

Observation of fluid microstructure variation contributes to the macroscopic properties variation analysis. The Cryo-environmental scanning electron microscope (Cryo-SEM, FEI, Hillsboro, OR, USA), which is widely used for microstructure observation, was employed in this work. A fluid sample was frozen at −185 °C and then sublimated before observation, keeping the structure of the fluid intact.

## 4. Results and Discussion

### 4.1. Characterization of DiPHAM

**Figure 1:**
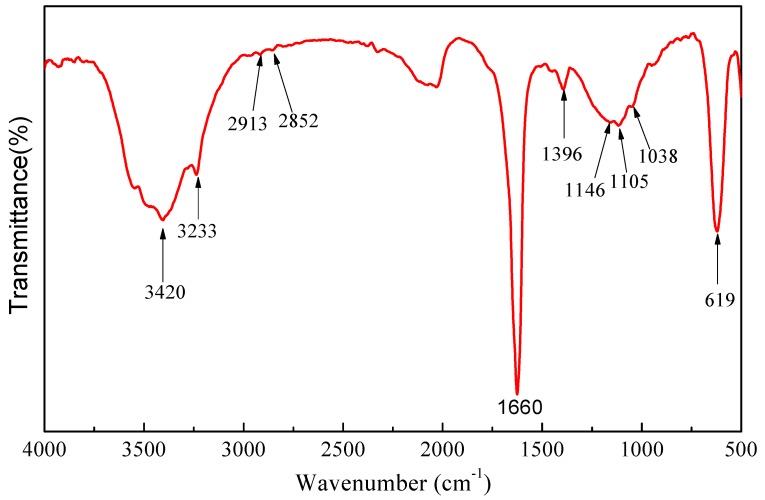
The FTIR spectrum of DiPHAM.

Figure 1 shows the FT-IR spectrum of DiPHAM. A very strong absorption band at 3420 cm^−1^ is due to the stretching vibration of –NH_2_ in the acrylamide groups (–CONH_2_). The band at 1660 cm^−1^ is due to the C=O stretching band, and the mixed surface bending vibration band of C-N is at 1396 cm^−1^. The band at 3233 cm^−1^ comes from the N-H stretching vibration. Bands at 1146 cm^−1^, 1105 cm^−1^, and 1038 cm^−1^ are assigned to characteristic symmetric and asymmetrical vibration absorption bands of –SO_3_^−^. The bands observed at 2913 cm^−1^ and 2852 cm^−1^ are assigned to characteristic symmetric and asymmetrical vibration absorption bands of –CH_2_^−^.The band at 619 cm^−1^ corresponds to the superimposed absorption band of the stretching vibration of –SO_3_^−^ and the out-of-plane bending vibration of –NH_2_ in –CONH_2_. The FT-IR spectra confirmed that DiC_12_AM and other monomers had been successfully introduced into the polymer.

**Figure 2:**
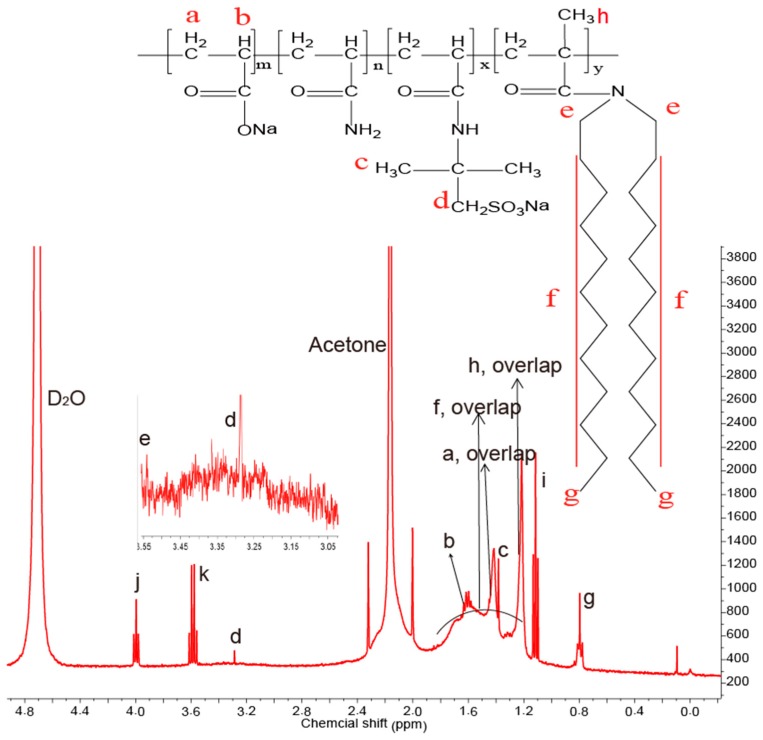
The ^1^H NMR (400 MHz, D_2_O) spectrum of DiPHAM.

Figure 2 showsthe ^1^H NMR (400 MHz, D_2_O) spectrum of DiPHAM. The resonances of protons are as follows: δ4.70 (D_2_O) for the solvent peak, δ1.47–1.56 ppm (a) for overlap-peaks, δ1.71 ppm (b) for the two protons resonances (–CH_2_-CH-) on the main chain, δ1.38 ppm (c) for the methyl proton peaks of the AMPS-Na group segment, δ3.29 ppm (d) for the methylene peak attached to the sulfonic acid foundation group of AMPS-Na, δ3.55 ppm (e) for the methylene peak attached to the tertiaryamine (C-N), δ1.20–1.90 ppm (f) for the methylene overlap-peaks of DiC_12_AM, δ0.80 ppm (g) for the methyl proton peak of DiC_12_AM, δ1.30 ppm (h) for the methyl proton overlap-peaks on the main chain, δ1.12 ppm (i) and δ3.56–3.61 ppm (k) for ethanol solvent peaks, δ3.98–4.01 ppm (j) for a solvent peak, and δ2.16 ppm for the acetone solvent peak. The results verified that the synthesized polymer was consistent with the designed DiPHAM.

### 4.2. Critical Association Concentration

**Figure 3:**
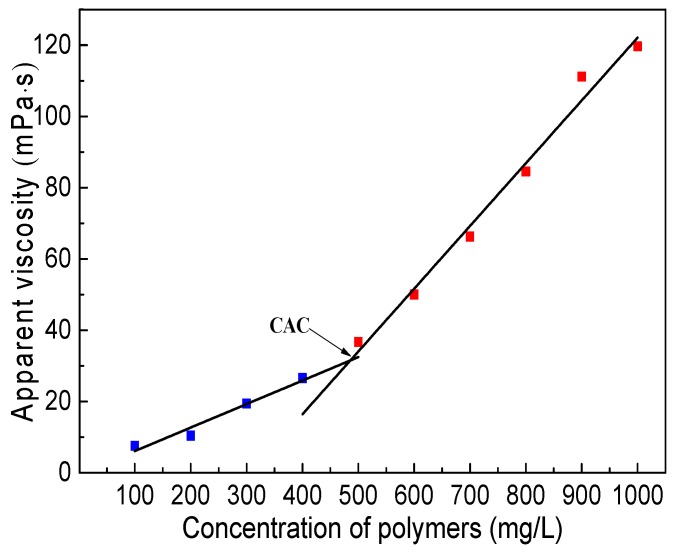
The critical association concentration (CAC) of DiPHAM.

The apparent viscosity of DiPHAM (7.34 s^−1^ shear rate) increases linearly with increasing concentration. However, the viscosity increases at different rates before and after the association of the polymer. Therefore, the intersection of the two lines in Figure 3 at 490 mg/L is assumed to be CAC. The CAC of DiPHAM, which is lower than common hydrophobic polymers (800–1500 mg/L), showed a strong hydrophobic effect, perhaps due to the symmetrical twin tailed hydrophobic chains introduced [18,19].

**Figure 4:**
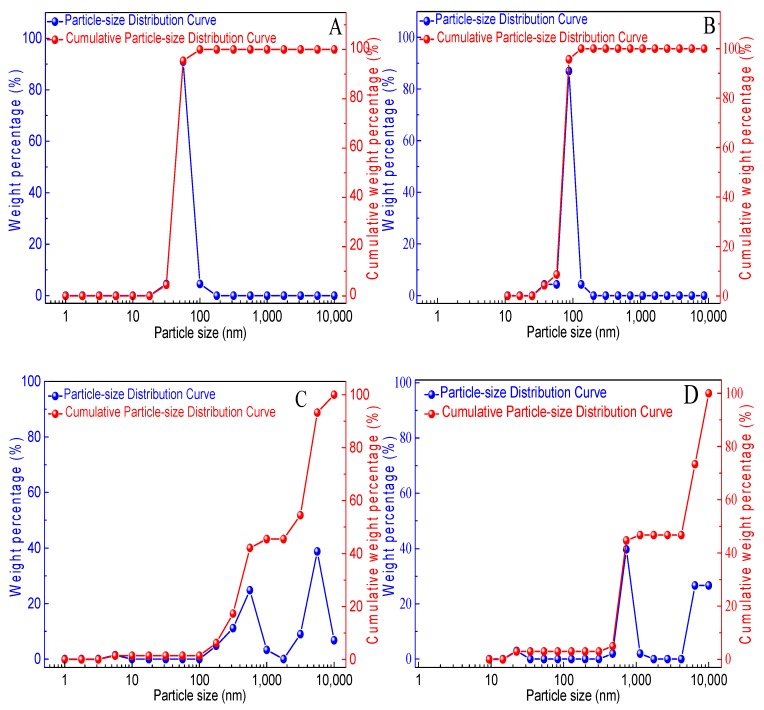
Size distribution of DiPHAM with different concentrations: (**A**) 100 mg/L (56.2 nm), (**B**) 300 mg/L (87.6 nm), (**C**) 500 mg/L (562 nm), and (**D**) 700 mg/L (732 nm).

**Figure 5:**
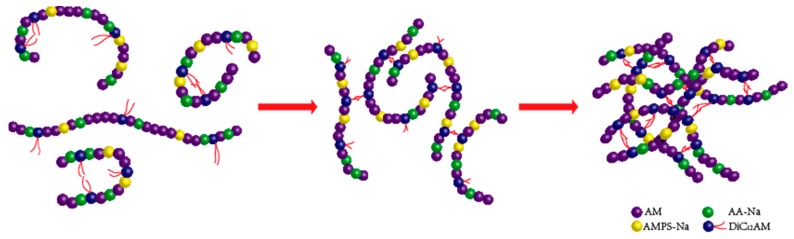
The association process of DiPHAM.

Various particle sizes (100–700 mg/L) in DiPHAM solutions were measured by DLS [20,21]. Figure 4 shows that the sizes of the polymer particles are 56.2 nm for 100 mg/L and 87.6 nm for 300 mg/L. Due to a large dispersion of the polymer in low concentration solutions, hydrophobic chains of the polymer were mainly in intramolecular association, and the particle sizes of the polymer are very small. The particle size of 562 nm in a 500 mg/L DiPHAM solution was much larger due to supramolecules forming via association and entanglement. It is clear that polymers were associated at this concentration. The particle size in a 700 mg/L DiPHAM solution is 732 nm, which shows that the scale of supramolecules increased. Increasing polymer concentration leads to a decrease in the distance between polymer molecules. Therefore, the hydrophobic chains begin to have a greater intermolecular association and form a dynamic physical cross-linked network, as shown in Figure 5. This leads to a sharp increases in fluid mechanical volume and viscosity [22].

**Figure 6:**
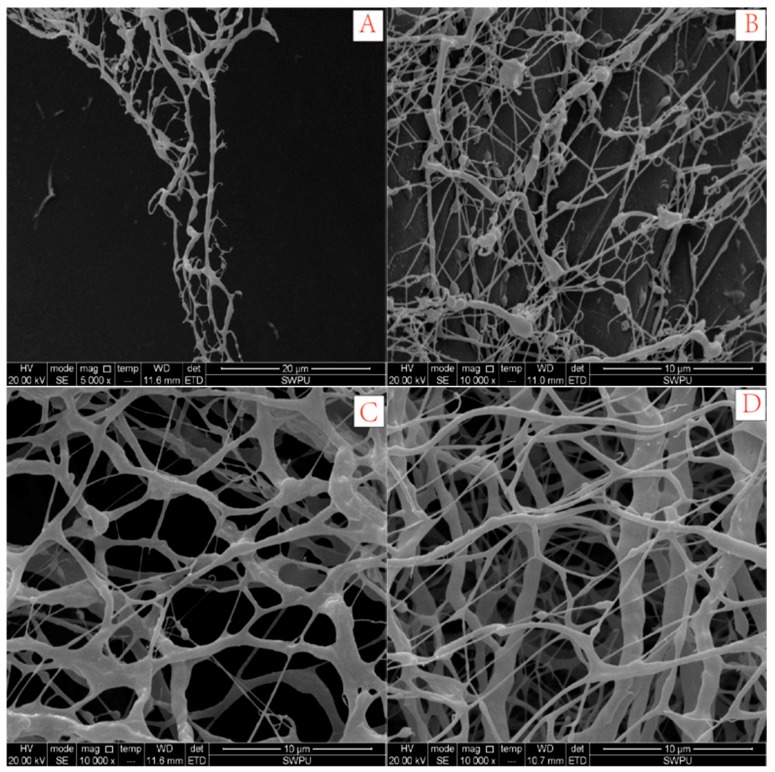
The representative SEM images of the DiPHAM aqueous solution association process. (**A**) 100 mg/L (×5000); (**B**) 300 mg/L (×10,000); (**C**) 500 mg/L (×10,000); (**D**) 700 mg/L (×10,000).

The SEM images shown in Figure 6 revealthe association process of DiPHAM in aqueous solution. The polymer was unable to form network structures at low concentrations, while association occurred at high concentrations (>CAC). Higher concentrations were related to closer association.

### 4.3. Effects of Metal Ions

**Figure 7:**
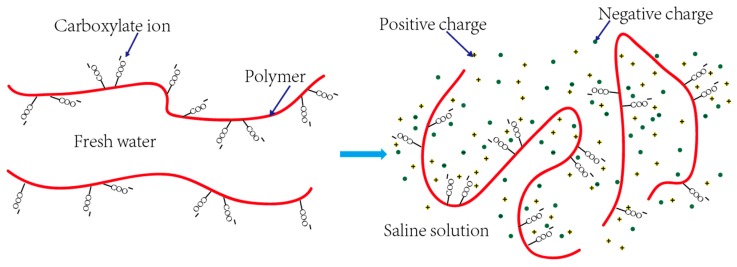
Electrostatic shielding of polymer in NaCl/KCl solution.

There are various mechanisms for the influence of metal ions, such as Na^+^, K^+^, Ca^2+^, and Mg^2+^, on the viscosity of conventional polymer solutions. The widely recognized mechanisms are electrostatic shielding and electric double layer compression of the polymer hydration shell [23]. In the modified polyacrylamide aqueous solution, acrylic acid groups can ionize to form negatively charged carboxylate ions (–COO^−^). Thus, the polymer chains extend farther into the aqueous solution because of the electrostatic repulsion among the carboxylate ions (Figure 7), resulting in a better thickening effect [24].

**Figure 8:**
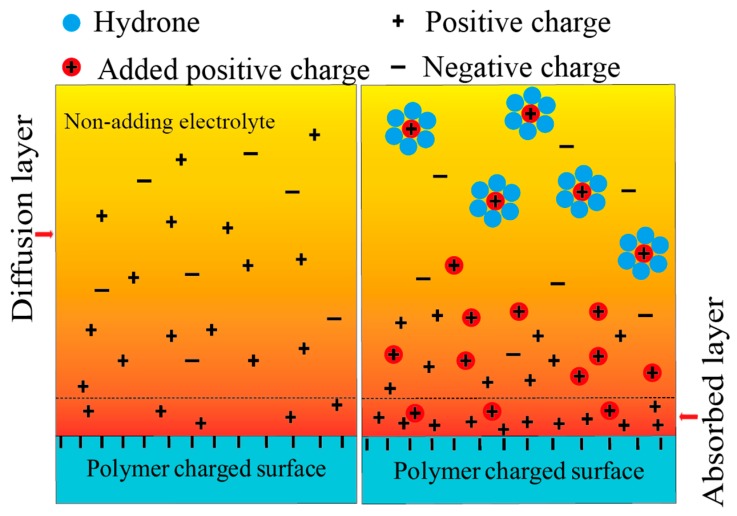
Compressing the thickness of the electric double layer of the polymer hydration shell.

The situation changes when metal ions are added. On the one hand, metal ions can weaken the electrostatic repulsion because of electrostatic shielding, allowing the polymer molecular chains to curl up. See the right image in Figure 7. This decreases the hydrodynamic volume and viscosity of polymer solutions [25]. The situation is different for divalent metal ions. At low metal ion concentrations, there is an increase in structural viscosity because two –COO^−^ share one Ca^2+^/Mg^2+^. Increased ion concentration in the polymer solution will cause the some of the original counter ions in the polymer chains’ diffusion layer to be squeezed into the adsorbed layer, as shown in Figure 8. This results in a decrease in thickness of the diffusion layer, correspondingly reducing the electric double layer potential and the mutual repulsion between molecular chains [26,27]. The decrease in distance between polymer chains due to the thickness decrease of the diffusion layer leads to an increase in attraction between them, which plays a dominant role in various molecular interactions [28,29]. These micro effects also reduce the extension of polymer molecular chains, decreasing the viscosity and the hydrodynamic volume of polymers.

In addition to the carboxylate ions in the modified polymer DiPHAM, to effectively weaken the effect of metal ions (such as Na^+^, K^+^, Ca^2+^ and Mg^2+^),AMPS, which is a salt-tolerant functional group, and DiC_12_AM, a strongly hydrophobic group, were introduced. AMPS is widely used as a salt-tolerant functional group in polymer design due to its strong anionic and water-soluble sulfonate moiety, which is insensitive to the attack of external ions [30,31]. A spatial network structure forms in DiPHAM aqueous solutions due to the intermolecular hydrophobic association. However, an increase in inorganic salt ion concentration increases the polarity of the solution, and the hydrophobic association action is enhanced. In addition, the electric double layer of the hydration shell around the hydrophobic groups is compressed by charged ions, which enhances the mutual attraction of hydrophobic chains. Therefore, the viscosity of DiPHAM aqueous solutions is increased in a way that enhances the hydrophobic association effect.

**Figure 9:**
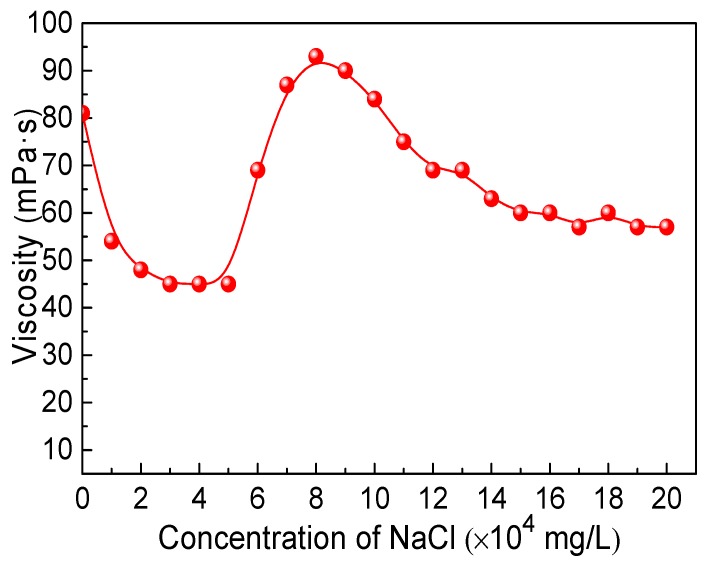
Effect of NaCl concentration on viscosity of a 0.3 wt% DiPHAM aqueous solution.

It has been reported that metal ions with different charge numbers have different effects on polymers in aqueous solutions [32,33]. In aqueous solution, Na^+^ and K^+^ are the most common monovalent ions, and their concentration is often relatively high. The most common divalent metal ions, Ca^2+^ and Mg^2+^, usually have lower concentrations in various water qualities. However, decades of research have revealed that small amounts of Ca^2+^ and Mg^2+^ tend to have a significant effect on polymer solutions [34]. In order to analyze the effects of different metal ions on the polymer aqueous solutions, we studied the effects of Na^+^, Ca^2+^, and Mg^2+^ on viscosity, hydrophobic association, and internal structure of polymer. Figure 9 shows the apparent viscosity curve of 0.3 wt% DiPHAM aqueous solution versus NaCl concentration. As the NaCl concentration increased, the viscosity of the DiPHAM aqueous solution initially decreased, followed by an increase, and finally decreased to a stable value of about 55 mPa·s.

**Figure 10:**
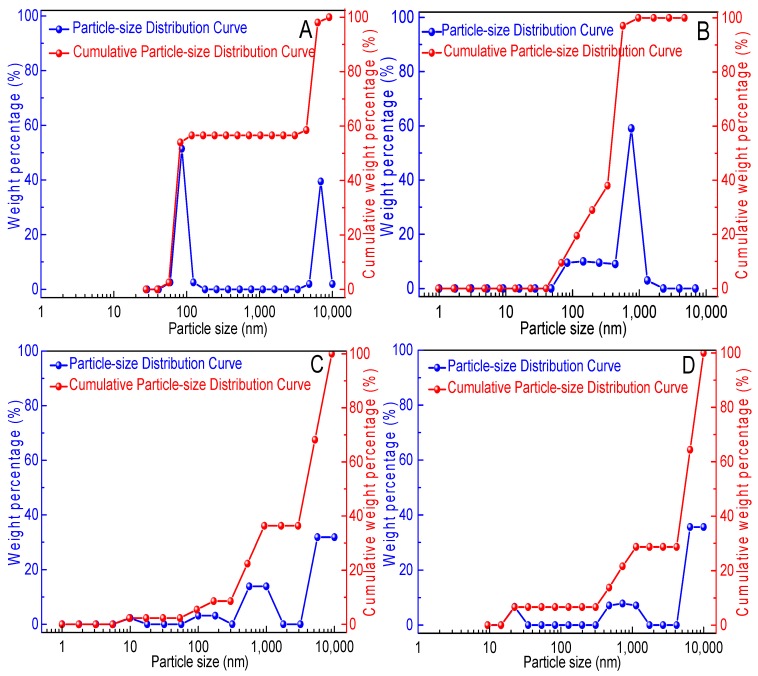
Particle size distribution of a 500 mg/L DiPHAM aqueous solution with different NaCl concentrations. (**A**) 4 × 10^4^ mg/L; (**B**) 8 × 10^4^ mg/L; (**C**) 12 × 10^4^ mg/L; (**D**) 16 × 10^4^ mg/L.

**Figure 11:**
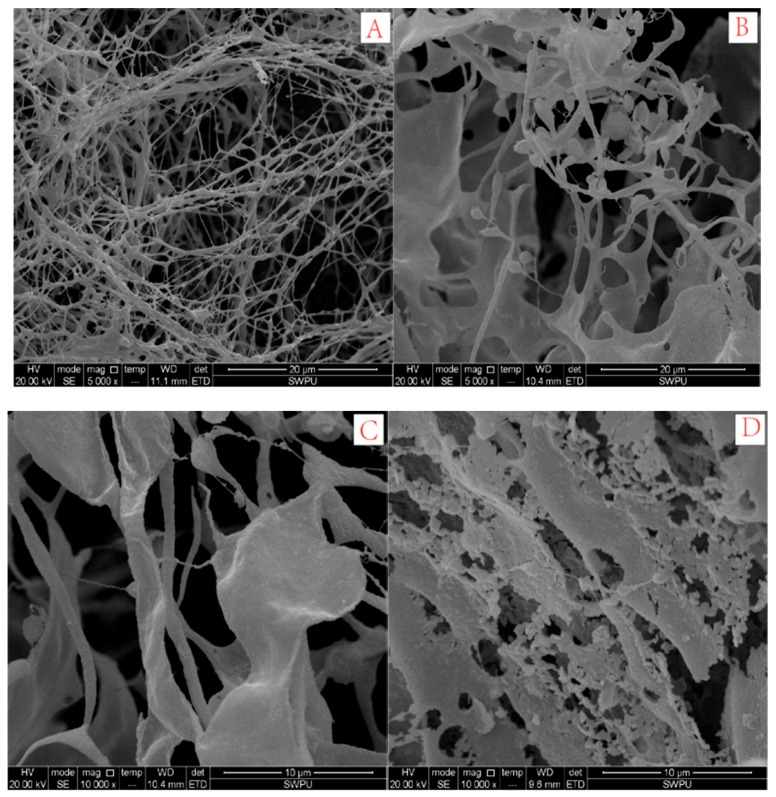
The representative SEM images of a 500 mg/L DiPHAM aqueous solution with different NaCl concentrations. (**A**) 4 × 10^4^ mg/L (×5000); (**B**) 8 × 10^4^ mg/L (×5000); (**C**) 12 × 10^4^ mg/L (×10,000); (**D**) 16 × 10^4^ mg/L (×10,000).

The varied aggregations of DiPHAM (500 mg/L) molecules in different NaCl concentrations (4 × 10^4^ to 16 × 10^4^ mg/L) were investigated by particle size measurement and SEM observations, and the results are shown in Figure 10 and Figure 11. For NaCl concentrations of 4 × 10^4^ mg/L, 8 × 10^4^ mg/L, 12 × 10^4^ mg/L and 16 × 10^4^ mg/L, the particle sizes were 85.82 nm, 758.23 nm, 562.63–5623.42 nm and 473.77–6468.38 nm, respectively, indicating an increase of particle size with increases in NaCl concentration. The particle size of 85.82 nm for 4 × 10^4^ mg/L NaCl is smaller than the particle size measured for a solution without NaCl (see Figure 4). The SEM images revealed the same result, and they showed that the spatial network structure is tighter. This indicated that the DiPHAM molecules were more crimped and less extended, resulting in a decreased viscosity. The particle size increased to 758.23 nm when NaCl concentration was 8 × 10^4^ mg/L, and the growth of the micelles and the formation of sheet-like structures can be observed from the SEM images. This indicates the strong hydrophobicity of the DiPHAM, increasing the contacts among polymer molecules and increasing the viscosity. Further increases in NaCl concentration led to greater increases in the particle sizes, which indicated enhanced hydrophobic associations of the DiPHAM, but the SEM images showed mass or sheet structures rather than network structures, which tends to diminish shear resistance. The increasing trend in particle size shows an important phenomenon of micro aggregation becoming smaller and macro aggregation gradually dominating, resulting in a continuously decreasing viscosity.

In conclusion, monovalent metal ions affect the viscosity of polymer solutions by electrostatic shielding and compressing the electric double layer of the polymer hydration shell. The decrease in viscosity caused by this can be represented by η_decrease_. However, hydrophobicity will be enhanced due to the increased polarity and the compression of the electric double layer of the polymer hydration shell caused by the added inorganic salts. This tends to increase viscosity, and this contribution to the viscosity can be represented by η_increase_ [35]. In the case of low metal ion concentrations, η_decrease_ is greater than η_increase_, and the viscosity of the solution goes down. With an increase in metal ion concentration, the hydrophobic association of the polymer molecules becomes stronger, η_increase_ becomes greater than η_decrease_, and the apparent viscosity increases. With further increased metal ion concentration, the polymer molecular chains will become more tightly packed due to the enhanced hydrophobic association. This results in a spatial structure change from a three-dimensional mesh space to a mass or sheet structure, and the viscosity of the polymer solutions decreases.

**Figure 12:**
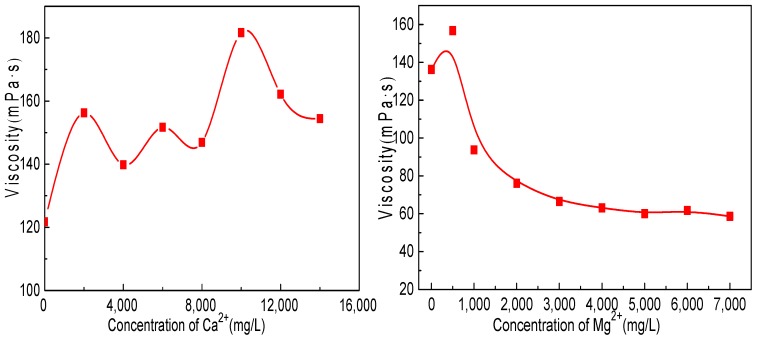
Effect of Ca^2+^/Mg^2+^ concentration on apparent viscosity of a DiPHAM aqueous solution.

Figure 12 shows the influence of divalent ions on the viscosity of a DiPHAM aqueous solution. The viscosity fluctuated with increasing of Ca^2+^ concentration, while for increasing Mg^2+^ concentration, the viscosity just increased slightly at first and then decreased steadily. It is clear that the higher-charged metal ions have a greater effect on polymer solutions than the lower-charged Na^+^ and K^+^ ions, and there is an obvious difference between Ca^2+^ and Mg^2+^ ions [36]. Divalent ions have the same influence mechanism as monovalent ions, but the compression of the electric double layer of the polymer hydration shell is enhanced. 

**Figure 13:**
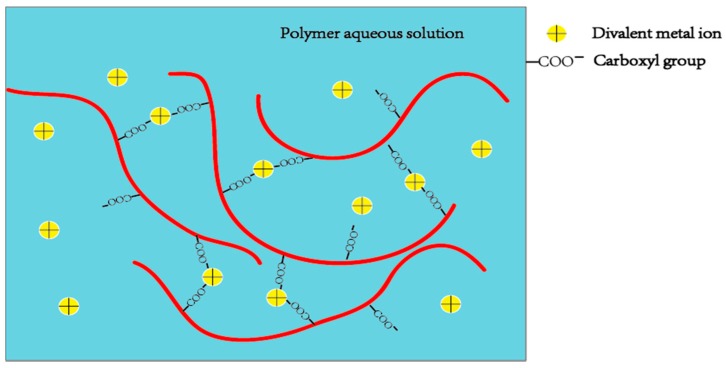
Solubility product balance among –COO^−^ groups and divalent metal ions in a polymer solution.

Figure 13 shows that there is a solubility product balance among –COO^−^ groups and Ca^2+^/Mg^2+^ ions that can lead to precipitation when the ion product is higher than the solubility product, and then the precipitate acts as a core and grows continuously [37,38]. Thus, –COO^−^ groups ionized from acrylic acid interact with Ca^2+^/Mg^2+^, producing a chemical bond force on the molecular chains. This enhances the structure strength, increasing the viscosity of the polymer solutions. Therefore, in a DiPHAM solution, increasing concentration of Ca^2+^/Mg^2+^ ions causes variations in viscosity due to the combination of the electrostatic shielding, compression of the electric double layer of polymer hydration shell, hydrophobic association enhancement, and the chemical bond action of the crystal core among molecular chains.

**Figure 14:**
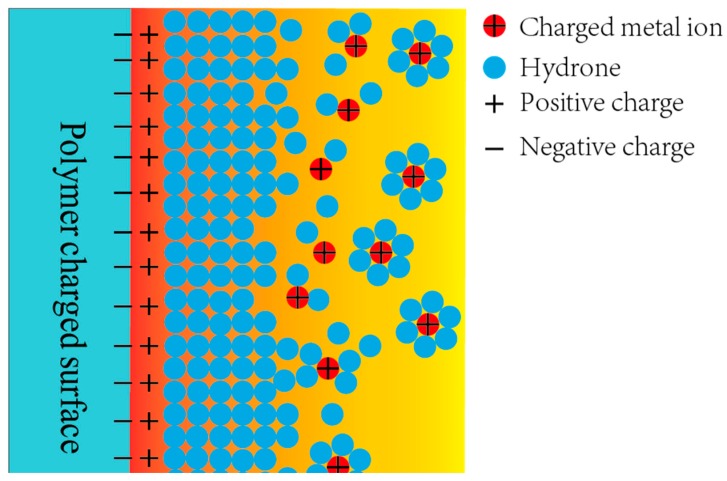
Reduction of the polymer hydration shell by metal ions.

The viscosity of a DiPHAM aqueous solution fluctuates because of changes in the relative extent of the influencing factors with changes in the Ca^2+^/Mg^2+^ ion concentrations. Although the charge of Mg^2+^ ions is the same as for Ca^2+^ ions, the ionic radius of a Mg^2+^ ion is smaller. This enhances the effect of Mg^2+^ on compression of the electric double layer of the polymer hydration shell [39]. There is a space around each dissolved ion where the ion has a significant influence on the water molecules. When strengths of the attractions between water molecules and ions are larger than the strengths of the hydrogen bonds between water molecules, water molecules tend to gather close to the ions and form a hydration shell, as shown in Figure 14 [40,41]. Due to the greater attraction between Mg^2+^ ions and water molecules than between Ca^2+^ ions and water molecules, the Mg^2+^ ions tend to gather more water molecules from the hydration shell near the polymer molecular chains. This thins the hydration shell, resulting in decreased extension of the polymer molecular chains [42].

### 4.4. Effect on Structure of DiPHAM

#### 4.4.1. Viscoelasticity Properties

The influence of metal ions on polymer structures can be observed by viscoelasticity measurements, because the viscoelasticity of polymers is closely related to the internal structure of the fluid. The investigations above have proved that Ca^2+^ and Mg^2+^ ions have a greater effect on polymer solutions than Na^+^ ions, so the disruptive effect of the Ca^2+^ and Mg^2+^ ions on the internal structure of a DiPHAM aqueous solution was studied by viscoelasticity measurement.

**Figure 15:**
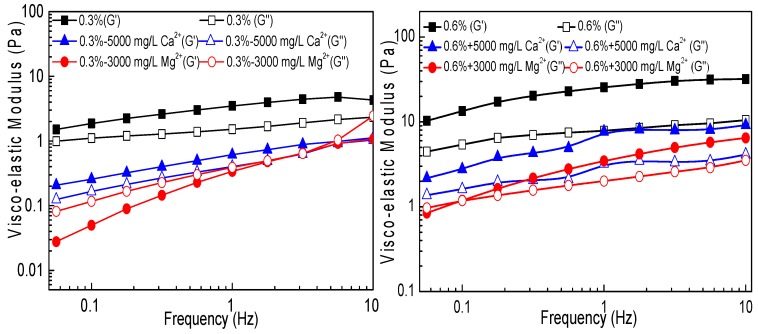
Viscoelasticity of a DiPHAM under the condition of high salinity.

The effects of Ca^2+^ and Mg^2+^ ions on the viscoelasticity of DiPHAM (0.3 wt% and 0.6 wt%) aqueous solutions were measured, and Figure 15 shows the results. It is clear that the modulus increased with increasing DiPHAM concentration, and the storage modulus (*G’*) was always greater than the loss modulus (*G”*) in the frequency range of 0.1–10 Hz. *G’* was always greater than *G”* for 5000 mg/L Ca^2+^ solutions, indicating that under this high-salinity condition, DiPHAM still has good viscoelasticity, and the structures of the polymer micelles have not been completely destroyed. For 3000 mg/L Mg^2+^, the storage modulus (*G’*) of the DiPHAM (0.3 wt%) solution was smaller than the loss modulus (*G”*). This indicates that Mg^2+^ ions cause more damage to the structure of DiPHAM aqueous solutions than Ca^2+^. However, increased DiPHAM concentrations improved this situation, even though the modulus still decreased, indicating that increased DiPHAM concentration can enhance the resistance to metal ions.

#### 4.4.2. Thixotropy

**Figure 16:**
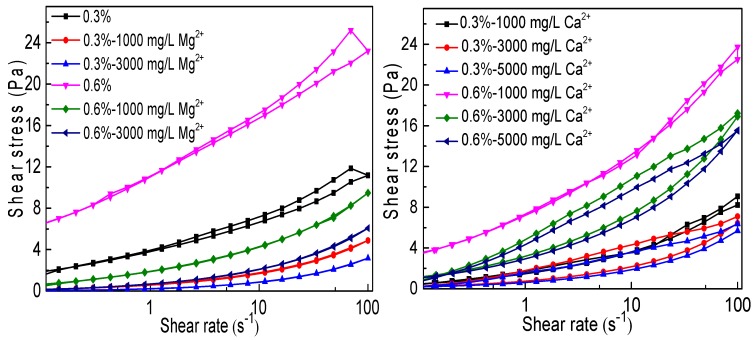
Thixotropy of different DiPHAM concentrations and with different concentrations of ions.

Thixotropy, which is reflected in the curve of shear stress with shear rate, is widely used to describe the internal structure changes of fluids under the influence of external forces [43,44]. Thixotropy measurements can help us understand the structure recovery of a fluid after the first structure disruption. The graphs in Figure 16 show that initially the shear rate gradually increased to a certain value (rising curve) and then decreased to the initial value (declining curve), forming a closed loop. For thixotropic fluids, the declining curve does not return to the initial value completely along the track of the rising curve, forming a hysteresis loop [45,46]. The larger the area is within the closed loop, the harder it is for the fluid structure to recover. In addition, the larger the area of the closed loop is, the more energy is needed to disrupt the structure [47,48]. The thixotropy variations of the aqueous polymer solutions correspond to the internal structure changes, and they reflect the effect of metal ions on DiPHAM solutions.

The shear stress of the DiPHAM aqueous solutions was recorded at a shear rate from 1 to 100 s^−1^, and then from 100 to 1 s^−1^. The recovery period was 5 min, and Figure 16 shows the results. It is clear that the increased DiPHAM concentration resulted in increased shear stress, which indicated increased structure strength or intramolecular friction. However, addition of the metal ions obviously weakened the shear stress, especially the Mg^2+^ ions. Moreover, the hysteresis loop areas decreased in the presence of Mg^2+^, which revealed the disruption of the structure of the DiPHAM in aqueous solution. Unlike the effect of the Mg^2+^ ions, when Ca^2+^ ions were added, the hysteresis loop areas increased (A_3000_ > A_5000_ > A_1000_), even as the shear stress decreased. This indicates that the structure strength increases under the high-salinity conditions of Ca^2+^. Furthermore, it is clear that the strength of the chemical bond between Ca^2+^ and –COO^−^ ions is stronger than that between Mg^2+^ and –COO^−^ ions. 

#### 4.4.3. Microstructure Observation

**Figure 17:**
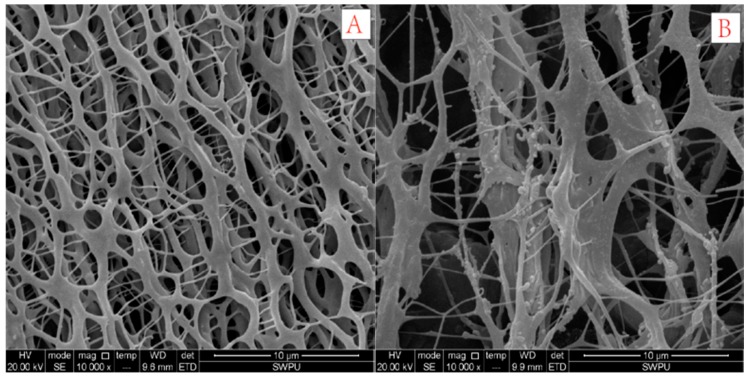

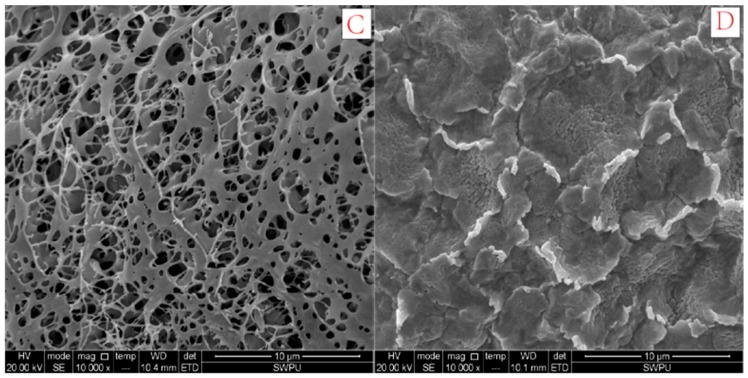
SEM images (×10,000) of a 0.3 wt% DiPHAM aqueous solution with different concentrations of Ca^2+^ ions (**A**) 1000 mg/L; (**B**) 3000 mg/L; (**C**) 5000 mg/L; (**D**) 10,000 mg/L.

**Figure 18:**
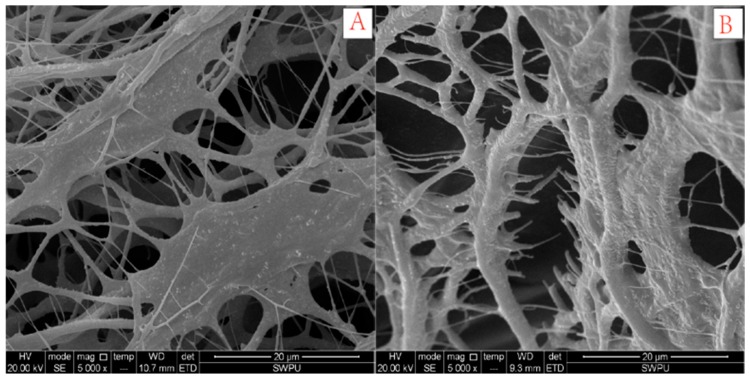
SEM images (×5000) of a 0.3 wt% DiPHAM aqueous solution with different concentrations of Mg^2+^ ions (**A**) 1000 mg/L; (**B**) 3000 mg/L.

SEM observation can help to describe the internal structure and strengths of attractions of DiPHAM with different concentrations of Ca^2+^/Mg^2+^ ions. The SEM images in Figure 17 show the influence of Ca^2+^ ions on spatial structures in 0.3 wt% DiPHAM aqueous solutions. The structure of the DiPHAM micelles gradually transforms from network to sheet-like with the increase of Ca^2+^ ions concentration, which shows the effect of the Ca^2+^ on DiPHAM micelles. Figure 18 shows the influence of Mg^2+^ ions on polymer micelle structures. DiPHAM aqueous solution with 1000 mg/L and 3000 mg/L of Mg^2+^ still maintained an integrated network structure. These SME images indicate that the DiPHAM has a good tolerance for Ca^2+^ and Mg^2+^.

### 4.5. Effect of Metal Ions on Rheological Properties

**Figure 19:**
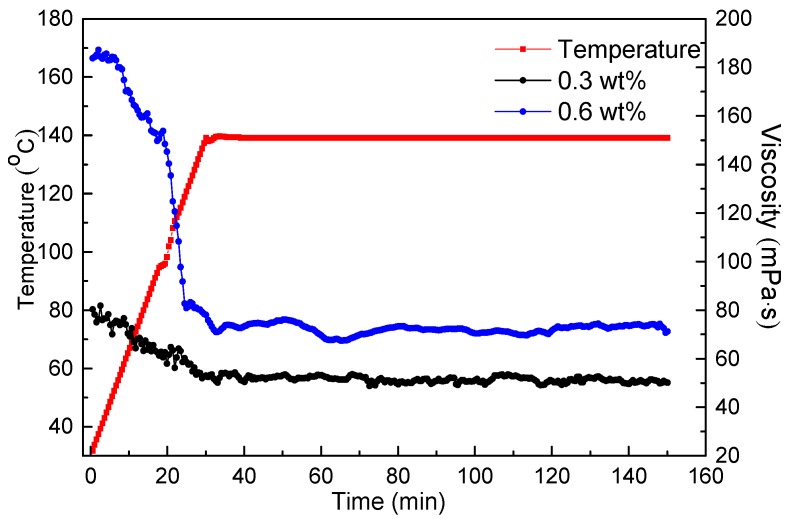
Rheological properties of 0.3 wt% and 0.6 wt% DiPHAM aqueous solutions at 140 °C.

The rheological properties of DiPHAM aqueous solutions were tested using a HAAKE MAR III RS 600 rheometer to investigate the possible application of these solutions in high-temperature and high-shear-rate conditions. In the measurement process, the temperature was increased from 30 to 140 °C within 30 min, and then the solutions were continuously sheared for 120 min at 170 s^−1^. Figure 19 shows the rheological properties for 0.6 wt% and 0.3 wt% DiPHAM aqueous solutions. The DiPHAM aqueous solutions showed good viscosities under the test conditions.

**Figure 20:**
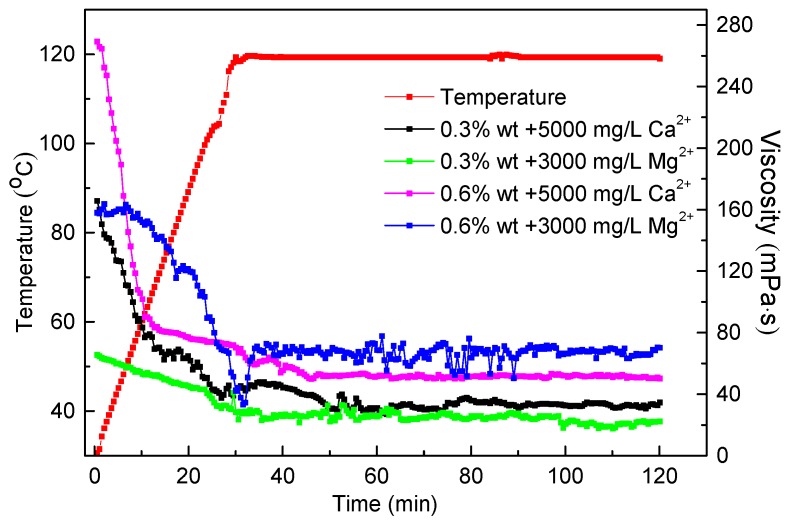
Rheological properties of 0.3 wt% and 0.6 wt% DiPHAM aqueous solutions with different concentrations of Ca^2+^ and Mg^2+^ ions.

Figure 20 shows the rheological properties of 0.3 wt% and 0.6 wt% DiPHAM aqueous solutions with salinity of 5000 mg/L Ca^2+^ and 3000 mg/L Mg^2+^ at 120 °C. The viscosity of 0.3 wt% and 0.6 wt% polymers with 5000 mg/L Ca^2+^ stayed at 35 mPa·s and 50 mPa·s, respectively, after 120 min of shearing. The viscosity of 0.3 wt% and 0.6 wt% DiPHAM with 3000 mg/L Mg^2+^ stayed at 22 mPa·s and 70 mPa·s, respectively. These results indicate that increasing the DiPHAM concentration can enhance the solution’s rheological properties under high-salinity and high-temperature conditions. Moreover, DiPHAM can be applied as a fracturing fluid thickener to improve oil and gas recovery under the high-salinity conditions of at least 5000 mg/L Ca^2+^ or 3000 mg/L Mg^2+^ [49,50].

## 5. Conclusions

The hydrophobic association polymer (DiPHAM) was synthesized by combining the twin tails hydrophobic monomer (DiC_12_AM) with AM, AA and AMPS. It was found to have good salt tolerance characteristics. The mechanism for the influence of NaCl on micelles structures in DiPHAM aqueous solutions includes an electrostatic shield and electric double layer compression of hydration shell, while the strong hydrophobic performance caused the DiPHAM solution to maintain a high viscosity in solutions of 2 × 10^5^ mg/L NaCl. In addition, Ca^2+^ and Mg^2+^ ions can attach to anionic groups on the DiPHAM chains, which helped DiPHAM solutions to maintain their internal structural integrity and keep a good viscoelasticity and thixotropy in 5000 mg/L Ca^2+^ or 3000 mg/L Mg^2+^ aqueous solutions. The influence of Mg^2+^ ions was stronger because of the relatively smaller ionic radius, which increased the compression of the electric double layer of the polymer hydration shell. DiPHAM aqueous solutions were found to have a good rheology at 140 °C and 170 s^−1^ shearing conditions. Under high-salinity (5000 mg/L Ca^2+^ or 3000 mg/L Mg^2+^) conditions, the solution with 0.6 wt% DiPHAM still maintained a high viscosity after continuous shearing for 120 min under the conditions of 120 °C and 170 s^−1^ shearing. The good salt tolerance of DiPHAM makes it useful for a variety of applications, including in fracturing fluids for enhanced oil recovery (EOR) and in sewage treatment.
